# First peripheral drug-eluting stent clinical results from China: 1-year outcomes of the Zilver PTX China study

**DOI:** 10.3389/fcvm.2022.877578

**Published:** 2022-09-27

**Authors:** Wei Ye, Tanja Böhme, Weiguo Fu, Changwei Liu, Xiaoming Zhang, Peng Liu, Jiwei Zhang, Yinghua Zou, Xinwu Lu, Aaron E. Lottes, Erin E. O'Leary, Thomas Zeller, Michael D. Dake

**Affiliations:** ^1^Vascular Surgical Department, Chinese Academy of Medical Sciences, Peking Union Medical College Hospital, Beijing, China; ^2^Department of Angiology, Universitaets-Herz-Zentrum Freiburg-Bad Krozingen, Bad Krozingen, Germany; ^3^Department of Vascular Surgery, Zhongshan Hospital, Fudan University, Shanghai, China; ^4^Department of Vascular Surgery, Peking Union Medical College Hospital, Chinese Academy of Medical Science and Peking Union Medical College, Beijing, China; ^5^Department of Vascular Surgery, Peking University People's Hospital, Beijing, China; ^6^Department of Vascular Surgery, China-Japan Friendship Hospital, Beijing, China; ^7^Department of Vascular Surgery, Renji Hospital Shanghai Jiaotong University School of Medicine, Shanghai, China; ^8^Department of IR and Vascular Surgery, Peking University, Beijing, China; ^9^Department of Vascular Surgery, Shanghai 9th People's Hospital, Shanghai JiaoTong University School of Medicine, Shanghai, China; ^10^Weldon School of Biomedical Engineering, Purdue University, West Lafayette, IN, United States; ^11^Cook Research Incorporated, West Lafayette, IN, United States; ^12^Department of Medical Imaging, The University of Arizona, Tucson, AZ, United States; ^13^Department of Surgery, The University of Arizona, Tucson, AZ, United States; ^14^Department of Medicine, The University of Arizona, Tucson, AZ, United States

**Keywords:** drug-eluting stent, paclitaxel-eluting stent, peripheral artery disease, peripheral vascular disease, superficial femoral artery

## Abstract

**Purpose:**

The benefit of using the Zilver PTX drug-eluting stent (DES) in superficial femoral artery (SFA) lesions has been demonstrated in multiple clinical studies. This prospective, multicenter study evaluated the 1-year safety and effectiveness of the DES for the treatment of femoropopliteal lesions in a Chinese patient population.

**Methods:**

Patients with a single *de novo* or restenotic SFA lesion ≤140 mm and a Rutherford classification of 2 to 4 were treated with the DES. The primary endpoint was primary patency assessed by duplex ultrasound at 1-year. Secondary endpoints included adverse events, event-free survival (EFS), and freedom from target lesion revascularization (TLR). Clinical outcomes included Rutherford classification, ankle-brachial index (ABI), and the walking impairment questionnaire (WIQ).

**Results:**

In this study, 178 patients with symptomatic peripheral artery disease were enrolled at nine institutions in China. The average lesion length was 79.0 ± 48.6 mm (range 14.8–245.4 mm) and 50.0% of lesions were total occlusions. The 1-year primary patency rate was 81.9%. Covariate analysis revealed that lesion length (*p* < 0.01) was the only significant factor for patency. No paclitaxel-related adverse events or amputations were reported. The 1-year rate for EFS was 94.9% and freedom from TLR was 95.5%. Through 1-year, treatment with the DES resulted in statistically significant improvement in ABI and WIQ scores compared with pre-procedure (*p* < 0.001). Clinical improvement of at least 1 Rutherford class was achieved in 142 of 174 patients (81.6%).

**Conclusion:**

This study showed promising short-term results for the treatment of SFA lesions with Zilver PTX DES in Chinese patients.

**Unique identifier:**

ClinicalTrials.gov, identifier: NCT02171962.

## Introduction

Endovascular therapy has become the standard technique for the treatment of most symptomatic femoropopliteal lesions (1). The combination of balloon or stent angioplasty and local administration of an antiproliferative drug in the form of the drug-coated balloon (DCB) and drug-eluting stent (DES) was an important development in endovascular therapy. Meanwhile, international guidelines include recommendations for using DES for the treatment of femoropopliteal lesions (2, 3).

The superiority of the Zilver PTX DES over percutaneous transluminal angioplasty (PTA) and provisional bare metal stent (BMS) was demonstrated in a prospective, multinational, randomized controlled trial over a long-term period of up to 5 years (4, 5). The DES was approved by the FDA for the treatment of *de novo* or restenotic symptomatic lesions in native vascular disease of the above-the-knee femoropopliteal arteries in 2012 (6). Moreover, the safety and effectiveness of the DES were confirmed in a multinational single-arm study including in-stent restenotic lesions (7). However, the benefit of treatment with the DES has not yet been investigated for Chinese patients and as such, this study aimed to evaluate the safety and effectiveness of Zilver PTX DES in femoropopliteal lesions in a Chinese patient population.

## Methods

### Study design

**Figure 1:**
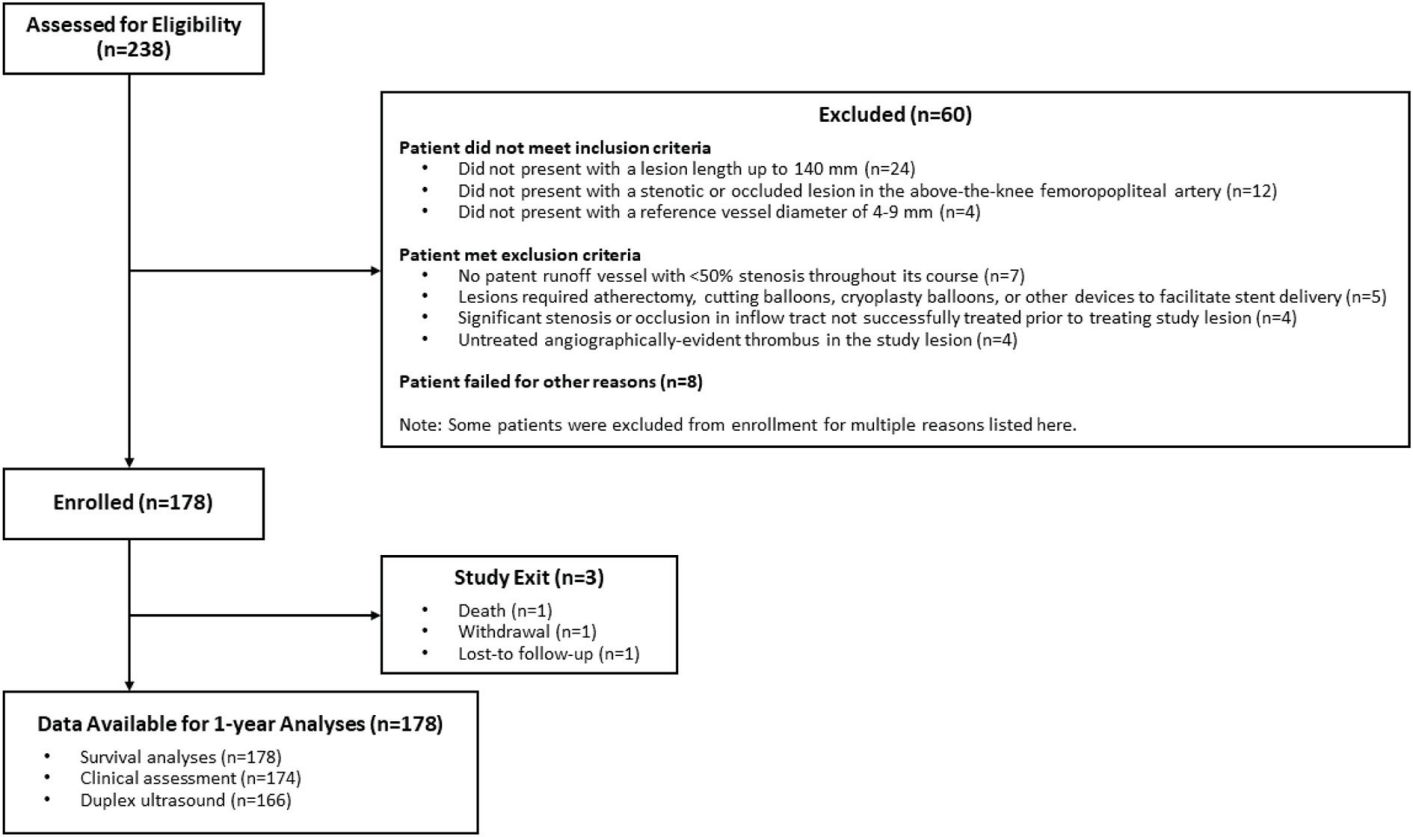
Patient flow diagram.

Between July 2014 and December 2015, this prospective study enrolled patients at nine institutions in China (ClinicalTrials.gov identifier NCT02171962). Patients had *de novo* or restenotic lesions of the above-the-knee femoropopliteal artery who met the inclusion and exclusion criteria. Key inclusion criteria included the following: Rutherford classification 2–4, >50% diameter stenosis (DS), minimum reference vessel diameter 4 mm, and lesion length ≤ 14 cm. Key exclusion criteria included: >50% stenosis of the inflow tract, no patent runoff vessel with <50% DS throughout its course, or previous stent in the study vessel. Figure 1 presents a patient flow diagram. Demographics, comorbidities, and baseline lesion characteristics are shown in Tables 1, 2. There was a 56.2% incidence of diabetes. By core laboratory measurement, the lesions had an average lesion length of 79.0 ± 48.6 mm (range 14.8–245.4 mm) and 50.0% were total occlusions.

**Table 1:** Patient demographics and comorbidities.

Patients, *n*	178
Mean age, years	67.4 ± 8.6 (45–89)
Male sex	78.8% (140)
Rutherford class 2	21.9% (39)
Rutherford class 3	70.2% (125)
Rutherford class 4	7.9% (14)
Diabetes	56.2% (100)
Type I	0% (0)
Type II	100% (100)
Hypertension	76.4% (136)
Hypercholesterolemia	18.5% (33)
Renal disease	5.6% (10)
Pulmonary disease	2.2% (4)
History of myocardial infarction	4.5% (8)
**Smoking status**	
Current	32.0% (57)
Past	25.3% (45)
Never	42.7% (76)

Continuous data are presented as the means ± standard deviation; categorical data are given as percentages (counts).

**Table 2:** Lesion characteristics.

Lesions, *n*	178
Lesion length, mm^a^	79.0 ± 48.6 (175)
**Lesion location**	
SFA	97.8% (174)
SFA/Popliteal	1.7% (3)
Popliteal	0.6% (1)
Previous intervention to study lesion	1.1% (2)
Occlusion^a^	50.0% (89)
Proximal RVD	4.8 ± 0.5 (178)
MLD in lesion, mm^a^	0.5 ± 0.7 (176)
Percent diameter stenosis, %^a^	88.5 ± 14.6 (176)
**Lesion calcification**	
None	24.2% (43)
Little	34.8% (62)
Moderate	31.5% (56)
Severe	9.6% (17)
**Patent runoff vessels^a^, ^b^**	
0	11.8% (21)
1	33.1% (59)
2	27.5% (49)
3	14.0% (25)
Not assessable	13.5% (24)

MLD, minimum lumen diameter; RVD, reference vessel diameter; SFA, superficial femoral artery.

Continuous data are presented as the means ± standard deviation; categorical data are given as percentages (counts).

a Angiographic core lab assessment.

b The site reported “0 patent runoff vessels” was 0%, as required per inclusion/exclusion criteria.

Patients provided written informed consent before the procedure, and approval was obtained from each site's institutional review board or ethics committee. Centralized core laboratories provided independent analyses of ultrasound images (VasCore; Boston, MA) and angiographic images (Beth Israel Deaconess Medical Center; Boston, MA).

### Baseline assessment, interventions, and medications

Rutherford classification, ankle-brachial index (ABI), and the walking impairment questionnaire (WIQ) were assessed pre-procedure. The drug-eluting nitinol stents (Zilver PTX Drug-Eluting Peripheral Stent, Cook Medical, Bloomington, IN) were 5 mm or 6 mm in diameter and 40 to 100 mm in length. The device instructions for use recommend that stents be oversized by 1 to 2 mm with respect to the reference vessel and placed at least 1 cm below the SFA origin and above the medial femoral epicondyle. Only a single lesion ≤14 cm in length per patient was permitted to be treated. Treatment of inflow and outflow disease using standard PTA was at the physician's discretion. Dilatation before stent placement was recommended. As in previous studies, the same antiplatelet regimen was recommended for all patients: clopidogrel 75 mg starting at least 24 h before the procedure or a procedural loading dose; continued clopidogrel 75 mg for at least 60 days post-procedure; and aspirin 100 mg indefinitely.

### Follow-up assessment and endpoints

Rutherford classification, ABI, and WIQ were assessed at the 6-month and 1-year clinical visits. The primary endpoint was primary patency at 1-year. Patency was assessed by duplex ultrasound (peak systolic velocity ratio [PSVR] ≤2.5) and/or angiography; in cases where both imaging modalities are available, angiography took precedence. Failure of primary patency of the study lesion occurred at the first instance of loss of patency (i.e., ≥50% DS, including the region within ±5 mm proximal and/or distal to the study segment), reintervention, total occlusion, surgical bypass due to restenosis, or amputation of the extremity due to restenosis of the study lesion. Additional measures through 1-year included adverse events, event-free survival (EFS), freedom from target lesion revascularization (TLR), and comparison of clinical outcomes (ABI, Rutherford classification, WIQ score). EFS was defined as freedom from device- or procedure-related major adverse events, including death, amputation, clinically-driven TLR, target limb ischemia requiring surgical intervention, or surgical repair of the target vessel; and freedom from worsening of the Rutherford classification by two classes (or to class 5 or 6). Clinically driven TLR was defined as reintervention performed for ≥50% DS within ±5 mm of the target lesion after recurrent clinical symptoms of peripheral artery disease (PAD).

### Statistical analysis

The study hypothesis was that 1-year primary patency in a Chinese patient population would be similar to a performance goal based on the primary patency rate from the Zilver PTX arm of the Zilver PTX multi-center randomized control trial. The sample size was calculated based on a one-sided exact binomial test, power of 0.9, and type I error rate of 0.05, which resulted in a sample size of 152 patients. To account for patient attrition and potential loss to follow-up, ~175 patients were planned to be enrolled.

The data were analyzed using SAS for Enterprise Guide 7.1 (SAS Institute Inc., Cary, NC). Continuous variables are reported as means and standard deviations, with *p*-values calculated using the standard *t*-test. Categorical variables are reported as counts and percentages, with *p*-values calculated using Fisher's exact test. As appropriate, the number of observations represented the number of patients, the number of treated lesions, or the number of treated limbs. Kaplan–Meier analyses were performed to assess patency, event-free survival, and freedom from TLR over time. A Cox proportional hazard model was performed using relevant covariates expected to have a potential impact on patency.

## Results

A total of 249 stents (mean of 1.4 stents per lesion) were implanted. The most commonly used stent diameter was 6 mm (64% of stents), and the most commonly used stent length was 100 mm (35% of stents). One patient was lost to follow-up and one patient withdrew from the study, and 1-year follow-up was obtained for 174/177 patients, representing 98% of those eligible.

**Figure 2:**
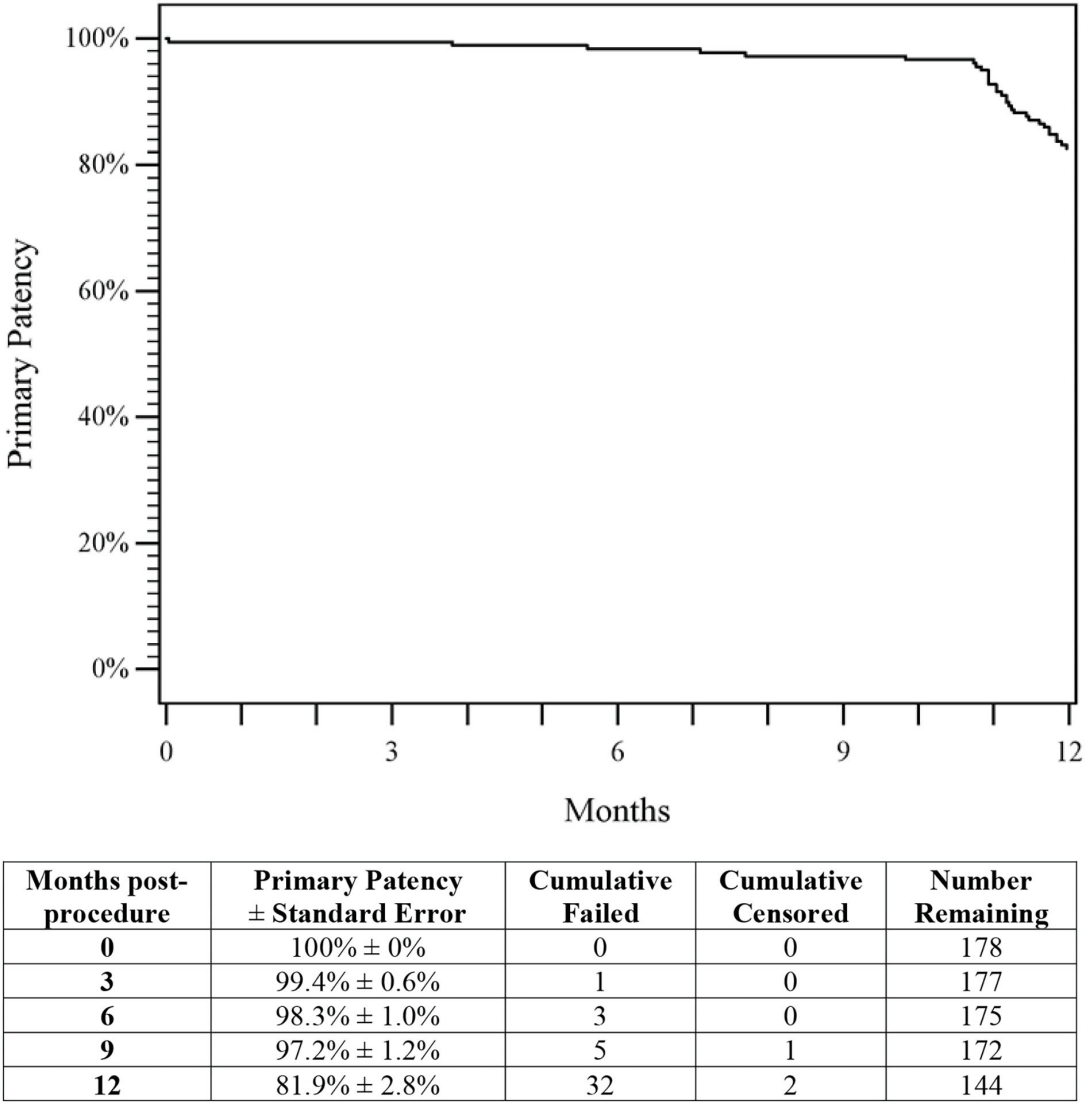
Primary patency. The Kaplan–Meier curve shows an 81.9% rate of primary patency through 1-year for patients treated with DES. The life table is included. DES, drug-eluting stent.

Duplex ultrasound was performed for 94% of patients. Based on Kaplan–Meier estimates, the 1-year primary patency rate was 81.9% (95% CI 76.4 to 87.5%; Figure 2). This rate is significantly greater than the performance goal of 72.7%, and the primary hypothesis is met (*p* = 0.003), demonstrating the effectiveness of the DES in maintaining vessel patency for a Chinese patient population. Duplex ultrasound-derived patency rates by stent size and site-measured vessel diameter are shown in Table 3. Covariate analysis revealed that lesion length (*p* < 0.01) was the only significant factor for patency (Table 4).

**Table 3:** Patency rates by stent size and vessel diameter.

	**5 mm stent**	**6 mm stent**
**RVD** **=** **4.0**	84% (*n* = 25)	100% (*n* = 1)
**RVD** **>** **4.0**	63% (*n* = 43)	89% (*n* = 109)

RVD, reference vessel diameter.

**Table 4:** Patency covariate analysis.

**Covariate**	***p*-value**	**Hazard ratio**
Lesion length (mm)	< 0.01	1.011
5 mm stent diameter	0.06	1.882
Proximal RVD (mm)	0.07	0.587
Rutherford classification	0.16	2.079
Current smoker	0.19	0.567
Patent runoff vessels	0.27	1.431
Renal disease	0.41	0.417
Male	0.47	1.352
Diabetes	0.56	1.225
Past smoker	0.57	0.770
Age (years)	0.57	0.990
Coronary artery disease	0.63	1.185
Total occlusion	0.79	0.910
Calcification	0.87	1.062

RVD, reference vessel diameter.

**Figure 3:**
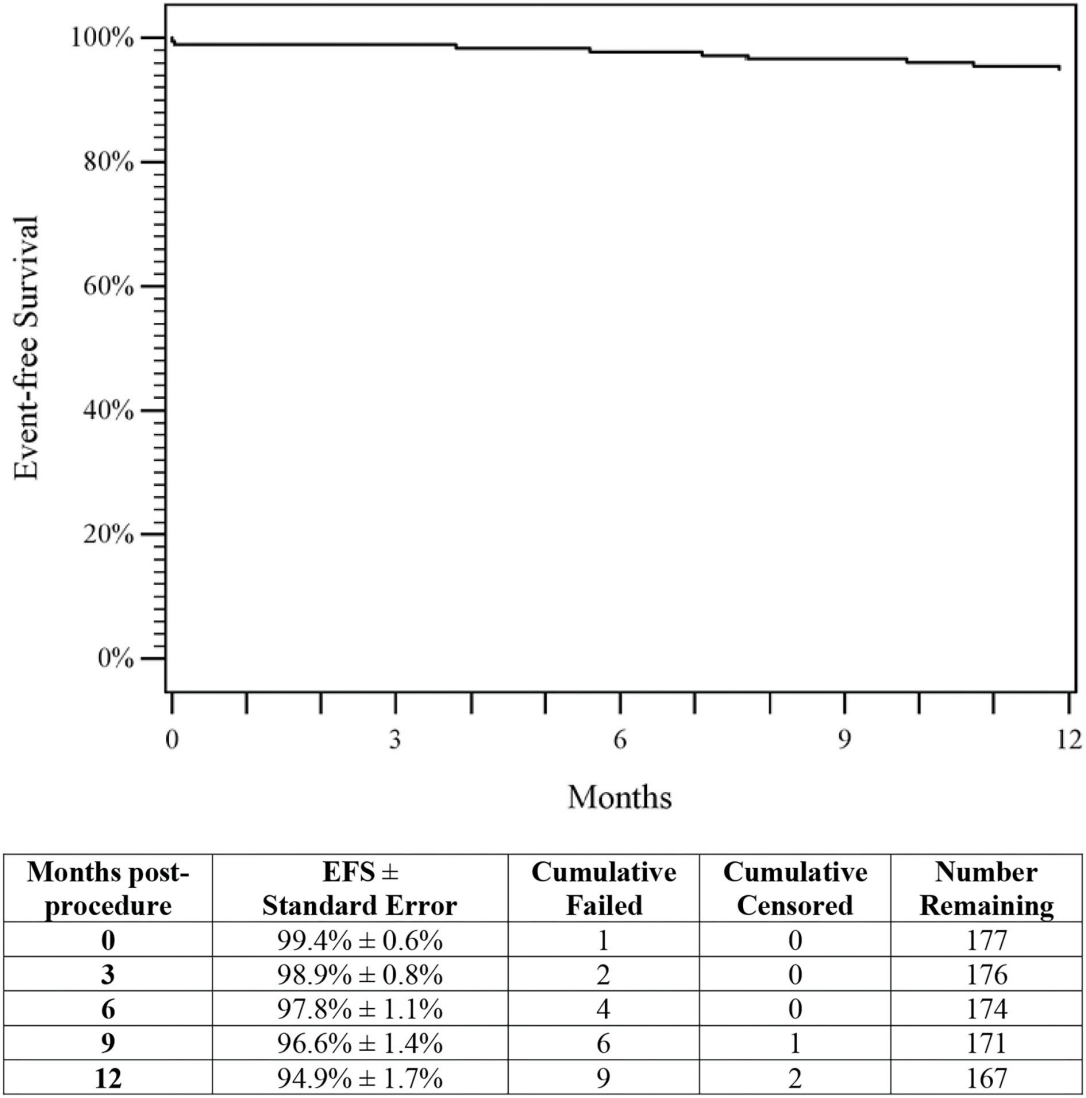
Event-free survival. The Kaplan–Meier curve shows a 94.9% rate of event-free survival (EFS) through 1-year for patients treated with DES. The life table is included. DES, drug-eluting stent; EFS, event-free survival.

There was a single death, due to a car accident, during this study; this event was not related to the study device or study procedure. No paclitaxel-related adverse events or amputations were reported. EFS through 1-year was 94.9% (95% CI 98.2 to 91.7%; Figure 3), with clinically driven TLR accounting for eight of the nine events, demonstrating the safety of DES in Chinese patients.

**Figure 4:**
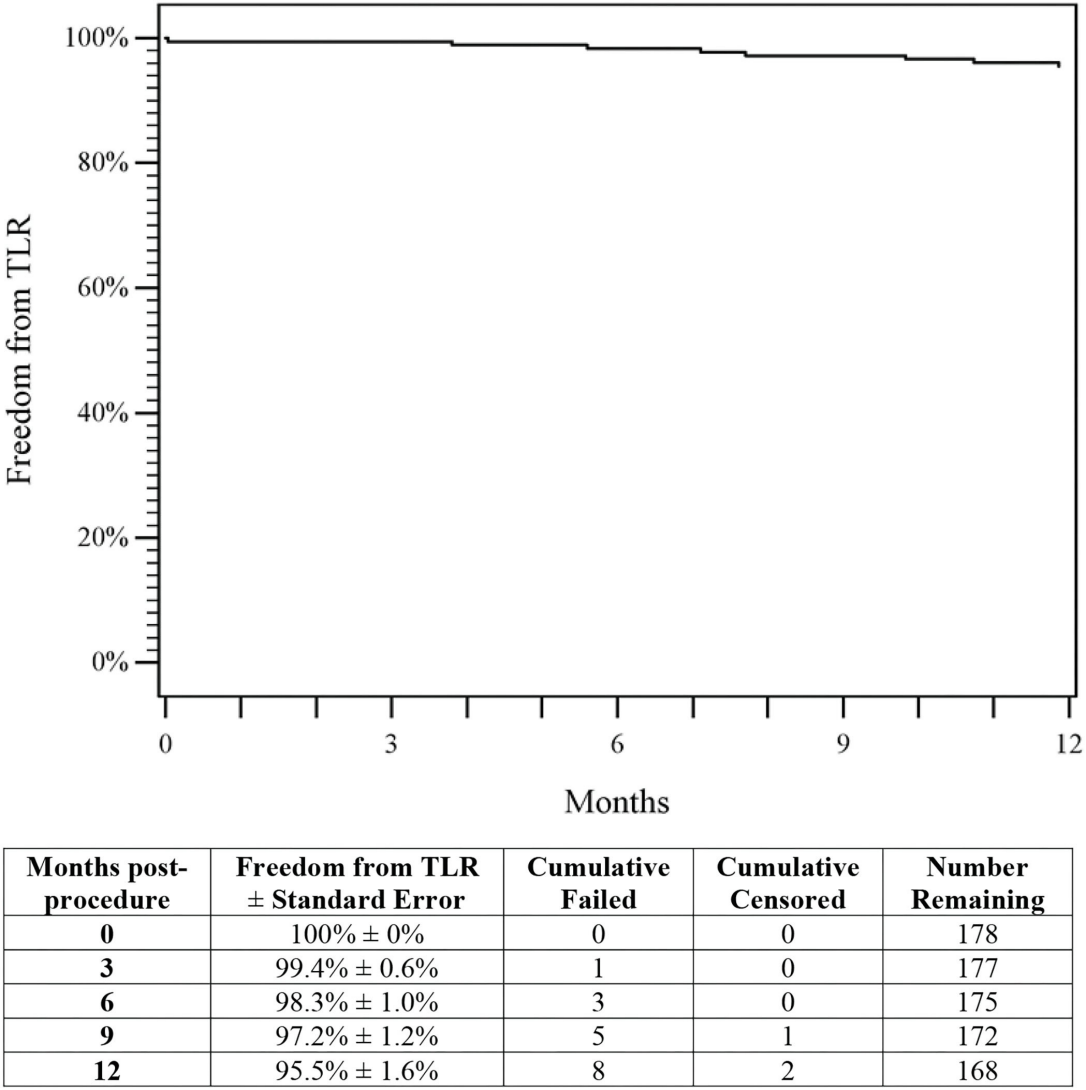
Freedom from TLR. The Kaplan–Meier curve shows a 95.5% rate of freedom from TLR through 1-year for patients treated with DES. The life table is included. DES, drug-eluting stent; TLR, target lesion revascularization.

**Figure 5:**
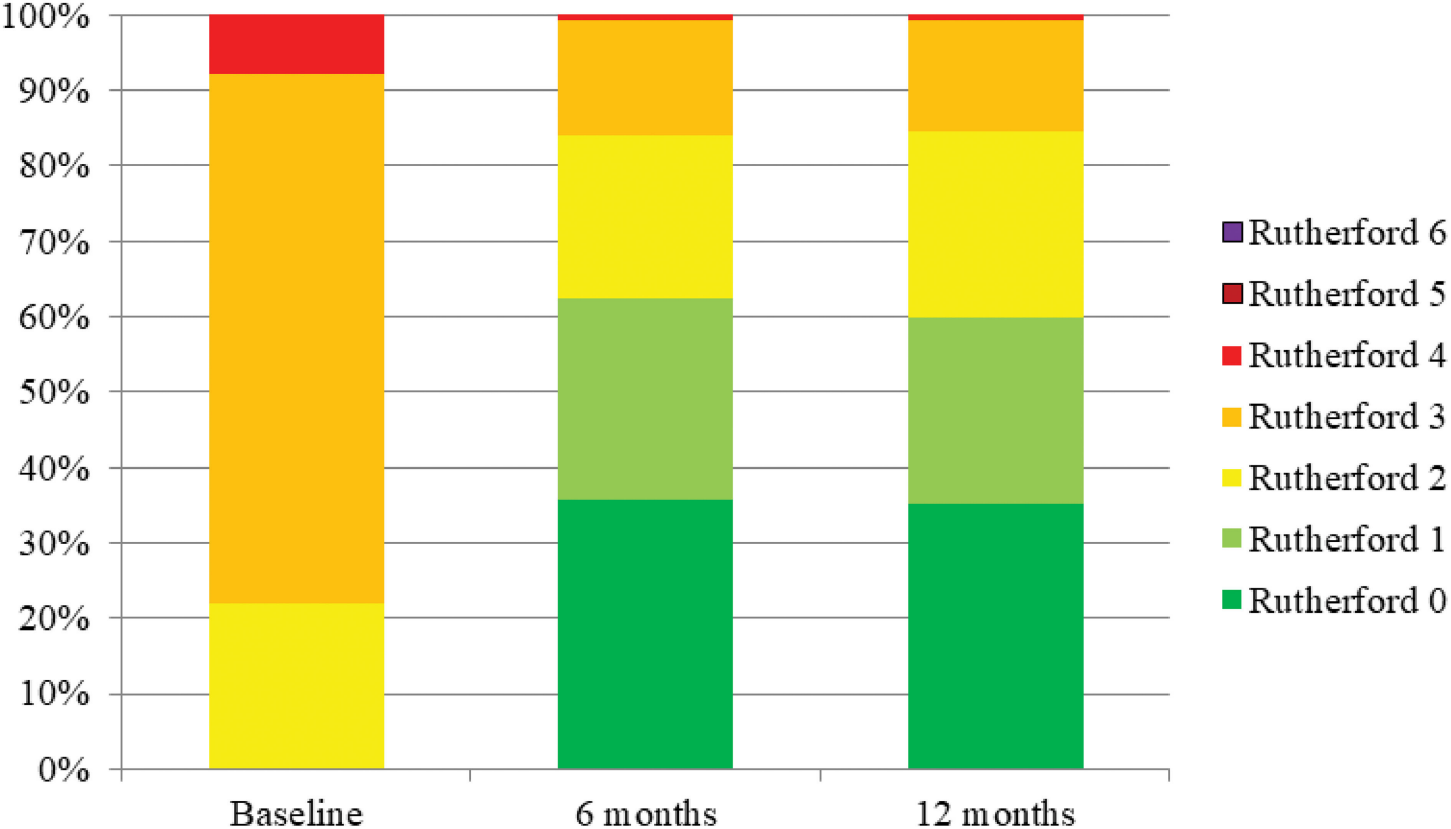
Rutherford classification. Rutherford classification significantly improved through 1-year compared to pre-procedure (*p* < 0.001).

The 1-year Kaplan–Meier estimate of freedom from TLR was 95.5% (95% CI 98.5 to 92.5%; Figure 4). Clinical assessment at 1-year revealed significant improvement in Rutherford classification (*p* < 0.001, Figure 5). Clinical improvement of at least 1 Rutherford class was achieved in 142 of 174 patients (81.6%), and 60% of patients were classified as Rutherford class 0 or 1 at 1-year. Similarly, ABI and WIQ scores also significantly improved at 1-year (*p* < 0.001, Table 5).

**Table 5:** Clinical outcomes.

**Clinical outcome**	**Pre-procedure**	**6 months^a^**	**1-year^a^**
ABI	0.59 ± 0.17 (*n =* 166)	0.87 ± 0.18 (*n =* 172)	0.83 ± 0.21 (*n =* 164)
WIQ distance score	22.9 ± 20.5 (*n =* 178)	74.3 ± 33.8 (*n =* 177)	77.9 ± 32.3 (*n =* 175)
WIQ speed score	27.8 ± 22.8 (*n =* 178)	52.0 ± 32.3 (*n =* 177)	52.5 ± 32.2 (*n =* 175)
WIQ climbing score	48.5 ± 33.8 (*n =* 178)	75.0 ± 33.5 (*n =* 172)	75.3 ± 35.3 (*n =* 173)

ABI, ankle-brachial index; WIQ, walking impairment questionnaire.

a p < 0.001 compared to pre-procedure; statistically significant.

## Discussion

The present study met the predefined effectiveness margin of the DES for the treatment of femoropopliteal lesions in a Chinese population. With a patency rate of 81.9%, the outcome was significantly greater than the performance goal of 72.7%, which was based on the 1-year outcome of the randomized controlled Zilver PTX trial (4).

Several studies have investigated outcomes after endovascular therapy of femoropopliteal lesions in Chinese patients. However, there are only a few randomized studies and comparability is difficult, as patients differ according to their risk factors and lesion characteristics. Prior studies describing the results for PAD patients after endovascular therapy have been published (8–10); however, these studies did not enroll Chinese patients which may limit the direct comparison of the results to the current study.

The superiority of using DCB for the treatment of femoropopliteal lesions in Chinese patients has been demonstrated. In the AcoArt I trial, 6-month angiographic and clinical outcomes were better after treatment with the paclitaxel-coated balloon than after treatment with an uncoated balloon (11). After drug-coated balloon angioplasty, primary patency was 76.1% and freedom from TLR rate was 92.8% through 6 months (11). High patency and low reinvention rates were also observed in the IN.PACT SFA China study. Through 1-year, the primary patency rate was 90.9% and the freedom from TLR rate was 97.1% in patients treated with the DCB (12). The 1-year results of the present Zilver PTX China study, with a patency rate of 81.9% and freedom from TLR rate of 95.5%, are in line with these Chinese DCB studies.

There is limited evidence for stent treatment in femoropopliteal lesions in the Chinese patient population. A study from Taiwan compared the self-expanding polytetrafluoroethylene-covered Viabahn stent to BMS in chronic total occluded long femoropopliteal lesions. Compared with the BMS group, the Viabahn group had significantly less in-stent restenosis (18.2% vs. 42.9%) and TLR (21.8% vs. 50.0%) (13). The results with the DES from the current study were considerably better, although the lesions were not as challenging as the lesions in the study from Taiwan.

So far, there is no other published data available for the treatment of femoropopliteal lesions with DES in Chinese patients. The Zilver PTX randomized trial showed the superiority of the DES compared to both PTA and BMS. In comparison to acutely successful PTA, the primary patency rate after 1-year (83.1% vs. 65.3%, *p* < 0.001) was significantly better (4). In a randomized comparison of DES and BMS following PTA failure, the 1-year patency rate for the DES was significantly better (89.9% vs. 73.0%, *p* = 0.01) (4). In the Japanese post-market study, 21.5% of patients had critical limb ischemia, the lesion length was long at 14.6 ± 9.6 cm, and 18.6% of lesions had in-stent restenosis (14). Nevertheless, the Japan study also showed good results regarding freedom from TLR (91.0%) and primary patency (86.4%) through 1-year (14). The present study showed comparable event-free survival, primary patency, and freedom from TLR results to previous Zilver PTX studies through 1-year despite differences in patient and lesion complexity. In addition, clinical durability with the Zilver PTX DES was demonstrated in the randomized trial as well as the Japan study through 5 years (5, 15).

In this work, the covariate analysis showed lesion length as the only predictor of restenosis. The relationship between lesion length and patency is well-known for almost all interventional modalities (16–19). After BMS placement, the patency decreased linearly with the lesion length (17). Both after treatment with DCB and after DES at least a trend toward a higher restenosis rate was observed in longer lesions (18). In the Zilver PTX single-arm study, a subgroup analysis of 135 long lesions (>15 cm) showed primary patency of 77.6% at 1-year compared with 83.1% for the entire cohort (19).

In addition to lesion length, smaller stent diameter and smaller reference vessel diameter were near significant predictors of restenosis. These two factors are important in appropriate vessel preparation and stent oversizing. Undersizing reduces endothelial wall shear stress, a known predictor for restenosis development in the long term. Oversizing a stent by a large amount can also increase wall stress by creating an inflammatory reaction and resultant neointimal hyperplasia. However, moderate oversizing seems important, particularly when using smaller stents as seen in this study (20). As described in the instruction for use, the stent size should be selected in such a way that the stent diameter is at least 1 mm larger than the reference diameter. The importance of this instruction can be seen in the patency rates by stent size and vessel diameter. In vessels with a reference vessel diameter of 4 mm, a 5-mm stent is a good choice, resulting in a patency rate of 84% in this study. In comparison, patency rates were lower (63%) when a 5-mm stent was placed in vessels with a reference vessels diameter >4 mm. Further, when 6 mm stents were placed in vessels >4 mm, the authors report a patency rate of 89%. Thus, reinforcing that a 6-mm stent should be used to provide adequate oversizing for vessels >4 mm, consistent with the instructions for use.

According to a meta-analysis published by Katsanos et al. (21), the authors suggest that there is increased mortality beyond 2 years after treatment with paclitaxel devices. A 5-year survival analysis of 139 Chinese patients treated with IN.PACT Admiral DCB showed no difference in mortality. Further, there was no correlation between paclitaxel exposure and mortality (22). Compared with treatment with a Zilver PTX stent, paclitaxel exposure is significantly higher after using an IN.PACT Admiral DCB. A patient-level analysis of the Zilver PTX randomized controlled trial and Zilver PTX Japan post-market surveillance studies showed no increased long-term all-cause mortality compared to uncoated devices (23). A mortality signal was also not observed in a retrospective Japanese study (24). Additionally, in the present study, as well as in the randomized and the Japanese post-market study, no paclitaxel-related adverse events were reported (5, 15, 23).

An analysis estimated the prevalence of PAD in China and predicts that the total number of Chinese people with PAD will increase by 40% between 2000 and 2020 (25). By 2020, the total number of patients suffering from PAD is expected to be 41.13 million (25). It is therefore important to evaluate the optimal therapy options for Chinese patients to keep follow-up interventions and associated costs low (25). The Zilver PTX DES offers an excellent 1-year clinical outcome with a TLR rate as low as 4.5% and could therefore be a cost-effective treatment solution for femoropopliteal lesions in symptomatic Chinese PAD patients.

This study is limited by its single-arm design lacking a comparator study control group. However, the study was prospectively designed with inclusion and exclusion criteria that were similar to those for the pivotal study, and the safety and effectiveness results from this single-arm study are comparable to the results from the pivotal study. Another limitation is the clinical follow-up is only through 1-year; 1-year primary endpoints are consistent with primary endpoints for other endovascular devices treating lesions in the femoropopliteal artery.

## Conclusion

This study showed promising short-term results for the treatment of femoropopliteal lesions with Zilver PTX DES in Chinese patients. As expected, lesion length is a predictor of the incidence of restenosis and adequate stent diameter is important for optimal patency results.

## Data availability statement

The datasets presented in this article are not readily available; however, Cook Research Incorporated (CRI) is fully committed to supporting the principles of responsible data sharing, including providing qualified scientific researchers access to deidentified, patient-level data from CRI clinical studies to conduct legitimate scientific research. Data underlying the results reported in this article will be made available for request immediately after publication and ending 5 years after publication. Requests to access the datasets should be directed to Cook Research Incorporated Policy on Access to Clinical Study Data at https://www.cookresearchinc.com/extranet/data-access.html and submit a complete research proposal to request data access. Additional study documents, such as the study protocol, will be shared as needed if the data access request is granted. A data sharing agreement will be executed for access to deidentified patient-level data.

## Ethics statement

The studies involving human participants were reviewed and approved by each site's Institutional Review Board or Ethics Committee. The patients/participants provided their written informed consent to participate in this study.

## Author contributions

AEL and EEO'L contributed to the analysis and interpretation of the data and drafted and critically revised the manuscript. All other co-authors participated in the acquisition of the data and critically revised the manuscript. All authors have provided approval of the final manuscript and agree to be accountable for the published work.

## Conflict of interest

AEL and MDD are paid consultants for Cook Medical. EEO'L is a paid employee of Cook Medical. TZ has received honoraria from Abbott Vascular, Veryan, Biotronik, Boston Scientific Corp., Cook Medical, Gore and Associates, Medtronic, Philips-Spectranetics, and Shockwave; consulted for Boston Scientific Corp., Gore and Associates, Medtronic, Veryan, Intact Vascular, Shockwave, Bayer, and Vesper Medical; received (institution) research, clinical trial, or drug study funds from 480 biomedical, Bard Peripheral Vascular, Veryan, Biotronik, Efemoral, Cook Medical, Gore and Associates, Medtronic, Philips, Terumo, TriReme, Shockwave, Med Alliance, Intact Vascular, and B. Braun; and has common stock in QT Medical. This study was sponsored by Cook Medical. The funder had the following involvement with the study: study design, collection of data, analysis and interpretation of data, decision to publish the data, and writing the publication. The remaining authors declare that the research was conducted in the absence of any commercial or financial relationships that could be construed as a potential conflict of interest.

## Publisher's note

All claims expressed in this article are solely those of the authors and do not necessarily represent those of their affiliated organizations, or those of the publisher, the editors and the reviewers. Any product that may be evaluated in this article, or claim that may be made by its manufacturer, is not guaranteed or endorsed by the publisher.
